# We-perspective on vision impairment: pathways between common dyadic coping and relationship satisfaction

**DOI:** 10.3389/fpsyg.2025.1628804

**Published:** 2025-11-05

**Authors:** Stephanie Alves, Katharina Weitkamp, Christina Breitenstein, Guy Bodenmann

**Affiliations:** ^1^HEI-Lab: Digital Human-Environment Interaction Labs, Lusófona University, Lisbon, Portugal; ^2^Clinical Psychology for Children/Adolescents and Couples/Families, Department of Psychology, University of Zurich, Zurich, Switzerland

**Keywords:** common dyadic coping, relationship satisfaction, we-ness, intimacy, we-disease, vision impairment

## Abstract

**Introduction:**

Common DC, how couples cope together with stress, may protect couples from relationship dissatisfaction in the context of vision impairment. However, the mechanisms through which common DC relate to couple satisfaction are underexplored. This study aimed to examine whether we-ness, intimacy, and perceiving vision impairment as a we-disease mediate the relationship between common dyadic coping (DC) and relationship satisfaction in the context of vision impairment.

**Methods:**

Ninety-nine individuals with visual impairment (IVI) and their spouses completed self-report questionnaires assessing DC, relationship satisfaction, intimacy, we-ness, and we-disease. An Actor-Partner Interdependence Mediation Model was performed.

**Results:**

Results showed that (1) higher levels of common DC were associated with higher intimacy and we-ness which, consequently, were associated with higher relationship satisfaction in both IVI and their spouses; and (2) the more IVI’ spouses engage in common DC, the more IVI perceived we-disease and, consequently, the less their spouses were satisfied with the relationship.

**Discussion:**

Couples facing vision impairment benefited from conjoint coping efforts as they seem to strengthen their sense of togetherness and intimacy. The adaptiveness of dyadic appraisals for couples’ adjustment should be further explored in view of unexpected results. Psychosocial rehabilitation sessions should include both partners and promote conjoint coping strategies to address challenges associated with vision impairment.

## Introduction

Vision loss is a disability or impairment with a high impact causing several limitations in personal, professional, and social domains, which severely restricts activities of everyday life (Heussler et al., 2016). Worldwide, over half a billion people are blind or have moderate to severe visual impairment and forecasts predict that the number will double over the next 30 years (Steinmetz et al., 2021). Vision impairment demands constant readjustment of the individual and their social environment, representing a chronic stressor (Magnus and Vik, 2016), which can impact the independence and limits autonomy (Brown and Barrett, 2011). Therefore, in close relationships, a readjustment of distribution of roles and tasks and reduced equity in pragmatic domains is characteristic compared to couples without this health issue, and could negatively affect the couple relationship, increasing marital dissatisfaction (Lehane et al., 2017). A better understanding of protective factors against couple relationship dissatisfaction in the context of vision impairment is therefore worthful to inform prevention approaches.

According to the Systemic Transactional Model (STM; Bodenmann, 2005; Bodenmann et al., 2016), visual impairment can be considered as a *we-stress* (Bodenmann, 2005) or *we-disease* (Kayser et al., 2007; Leuchtmann and Bodenmann, 2017), as it concerns both partners directly and indirectly, having a pragmatic and emotional impact on both, but also requiring dyadic coping from both. Common DC is defined as efforts that both partners take together when facing a shared stressor in an attempt to solve problems together (*problem-oriented common DC*: joint information seeking, joint planning, joint problem solving) or joint emotion regulation (*emotion-oriented common DC*: calming down together, mutual encouraging, believing in the strengths of the couple, validating of previous efforts and shared reframing). Common DC turned out to be a powerful predictor of relationship satisfaction in community sample couples (Falconier et al., 2015), as well as among couples dealing with cancer, especially in comparison with other forms of DC (Ştefǎnuţ et al., 2021).

In the context of disability, Bertschi et al. (2021) summarized similar findings regarding common DC, however, mostly on the basis of qualitative studies. Specifically in couples dealing with one partner’s sensory impairment, common/joint DC might play a particular role as the couple as a unit faces stressors related to the impairment and both partners must deal with the demands related to this health issue (Breitenstein, 2021). Albeit delegated and supportive DC could play an important role in couples dealing with vision impairment on a practical level (e.g., doing things for the partners with visual impairment, supporting them in everyday activities and life management), the impairment itself is assumed to be dealt with more on a dyadic level by means of common/joint DC (Mamali et al., 2020). A recent study with couples facing one partner’s vision impairment supports this assumption, by demonstrating that common DC was the most relevant form of DC for couples’ psychological adjustment above and beyond supportive and delegated DC strategies (Alves et al., 2024). The authors argued that the predominant role of common DC over traditional forms of support could be explained by couples being relatively old and in long-term relationships, which may strengthen the couple ability to rely on conjoint DC strategies when dealing with dyadic stressors. Accordingly, a deeper examination of the contribution of common DC for couples’ relationship satisfaction in the context of vision impairment, as well as the mechanisms through which this relationship may occur, is worthful.

Little is known about processes (mediators) linking common DC and couple relationships. According to the STM, the association between common DC and relationship satisfaction may be explained by two mechanisms: shared coping leads to (a) reduced stress (the proverbial “a sorrow shared is a sorrow halved”) and is associated with a lower level of general stress in the couple (Bodenmann, 2005). This is going along with (b) fostering feelings of *we-ness* and togetherness, and an increase of mutual trust and intimacy (Bodenmann, 2005; Cutrona, 1996) as well as dyadic resilience (Kayser et al., 2007), which are viewed as the most important function of DC (Bodenmann, 2005; Cutrona, 1996). This assumption is also shared by the communal coping theory (Helgeson et al., 2018), which posits that successfully coping together with stressors may enhance the sense of teamwork within the couple and help to foster a stronger bond between partners. This study focuses on three potential mediators of the association between common DC and relationship satisfaction—we-ness, intimacy, and we-disease—which have similarities and theoretical overlaps, but still form distinctive constructs and emphasize different aspects.

We-ness (Reid et al., 2006) builds on theoretical approaches such as including the other in the self (Aron et al., 1991), cognitive interdependence (i.e., the extent to which individuals perceive the relationship as central to the self; Agnew et al., 1998), couple identity (i.e., partners’ capacity to view themselves as part of a union; Acitelli et al., 1999) or interdependence in stress experience as described in the STM (i.e., one partner’s experience of stress affects the other partner; Bodenmann, 2005; Bodenmann et al., 2016). Based on these theories, Topcu-Uzer et al. (2021) conceptualized we-ness as the extent to which partners perceive themselves as a unified entity (“we/us”), rather than two separate individuals (“I/me” or “you/him/her/they”) regarding their cognitions (sharing a similar understanding of life, vision, and perspective), emotions (feeling in synchrony, missing each other when absent, emotional availability to the partner), and behaviors (cooperation and sharing time together). Accordingly, it refers to a perceived sense of unity and shared identity, in which dependency and autonomy co-exist, allowing each partner to maintain individuality (Reid and Ahmad, 2015; Reid et al., 2006). It also includes a sense of togetherness and a mutual investment in, and commitment to, the relationship (Gildersleeve et al., 2017).

Aligned with theoretical assumptions, empirical research has found that common DC positively predicted couples’ sense of we-ness in couples from the community (Aydogan et al., 2024). In turn, a sense of we-ness has been found to be significantly related to relationship satisfaction among community samples (Acitelli et al., 1999; Cruwys et al., 2022; Özgülük Üçok et al., 2024; Reid et al., 2006), including emerging adults (Özgülük Üçok et al., 2025). Greater use of “we-talk,” a verbal indicator of we-ness, has been shown to protect couples with preschool-aged children from declines in marital satisfaction over time (Ouellet-Courtois et al., 2023). Moreover, recent cross-sectional studies highlighted the mediating role of we-ness in linking DC and relationship outcomes, such as relationship satisfaction in couples from the community (Nobandegani, 2022) and health-related dyadic processes (e.g., partner’s HIV-specific support) among couples dealing with chronic health conditions (HIV; Fu et al., 2025).

Intimacy also plays an important role in the process of dyadic stress management (Bodenmann, 2005) and support (Cutrona, 1996). Under stress, people feel safe when they know they can count and rely on their partner and their support. This feeling creates trust and goes hand in hand with the appraisal of being carried for, of being important to the other person and of not being abandoned in times of need. This experience allows for the development of secure attachment and intimacy. Two processes lead to intimacy according to the empirically tested model by Laurenceau et al. (1998): (a) mutual stress disclosure (i.e., stress communication in STM) and (b) perceived partner’s responsiveness (i.e., dyadic coping reactions in STM). In the context of vision impairment, we assume that partners who share their feelings, thoughts and worries about the impairment, support each other or engage in common DC (Bertschi et al., 2021) and therefore feel close to each other and share a particular intimacy that may even go beyond the one experienced by couples without this experience. This, in turn, should lead to greater relationship satisfaction, as suggested by evidence linking intimacy and relationship satisfaction (e.g., Rusbult and Buunk, 1993).

Despite some overlapping between we-ness and intimacy (e.g., both involve a sense of togetherness and emotional connection), a cognitive dimension (shared identity/unit) is more central in we-ness (Cruwys et al., 2022), strongly emphasizing the interdependence of behaviors, emotions, and cognitions between partners (beyond mutual disclosure and responsiveness; Gildersleeve et al., 2017). On the other hand, intimacy could be present without necessary perceiving a relational identity or mutuality (even though the relationship is close and trustful), and is more dependent on mutual disclosure-responsiveness interaction patterns between partners (Laurenceau et al., 1998). For instance, older couples are likely to perceive a stronger sense of we-ness than younger ones (Seider et al., 2009), even though often experience few helpful intimate disclosures over time (Jensen and Rauer, 2015). Accordingly, even though we-ness and intimacy can theoretically reinforce each other, we could argue that they entail more parallel processes, not necessarily depending on one another.

We-disease, on the other hand, is a concept related to major stressors, typically severe illness (e.g., cancer), impairment (e.g., sensory loss, physical handicap) or psychological disorders (e.g., depression), reflecting a notion of being affected by the stressor together (both are suffering from the health issue; Leuchtmann and Bodenmann, 2017). This concept was first introduced in the context of couples coping with cancer (Kayser et al., 2007) to describe the extent to which individuals appraise the patient’s illness as a shared problem (“we-disease”) rather than belonging solely to the patient. It could be viewed as one specific form of we-ness in the context of chronic illness, characterized by a sense of unity, togetherness and shared responsibility and mutual involvement for managing the health condition (Berg and Upchurch, 2007; Kayser et al., 2007).

However, while we-ness entails a broad perception of the couple as a unit, likely to be present in everyday life and less dependent from major stressors (Reid et al., 2006; Topcu-Uzer et al., 2021), we-disease is a situational appraisal that arises when couples emotionally and cognitively reframe one’s illness as “our problem” (Berg and Upchurch, 2007; Kayser et al., 2007). Furthermore, we-ness has been more examined in general close relationships research, while we-disease has mostly been studied in illness contexts. From a clinical standpoint, a we-disease orientation challenges the view that one partner is the patient and the other the supporting counterpart, rather, it implies that both partners are equally affected by the health condition, both are suffering, and thus both are committed to coping with it. This assumption has received large empirical evidence, mostly in samples of couples facing cancer, showing that not only the patient, but also the partner, might present poor psychological and social functioning, and that both partners’ adjustment is interrelated (Leuchtmann and Bodenmann, 2017). Also, couples with a shared disease appraisal are more likely to engage in joint coping efforts which, in turn, translate into higher relationship satisfaction, as recently demonstrated in a study with HIV serodiscordant male couples (Hou et al., 2025).

Since common DC strengthens partners’ emotional connectedness (Bodenmann et al., 2016), it could be expected that engagement in conjoint efforts of coping may thus contribute to fostering a we-perspective on the health impairment. For instance, when couples cope with stress together this may facilitate couples sharing personal experiences, including communicating about the impairment with each other, thus helping to strengthen dyadic appraisals of the impairment more closely (Bertschi et al., 2021), while their roles as patients and partners are de-emphasized (Helgeson et al., 2018). This is in line with communal coping theory (Helgeson et al., 2018), which posits that when couples cope together with health-related stressors, they come aware that this challenge is just one of many they may encounter in the future, and that each of these challenges can be faced together as a couple. Conversely, when couples dealing with chronic health problems mutually withdraw or disengaged from mobilizing common resources to manage dyadic stressors, they are more prone to feel disconnected from one’s partner, and their we-perspective be weakened (e.g., O’Keeffe et al., 2020). However, so far, this assumption has not been examined, nor the role of we-disease in the context of vision impairment. While in the context of cancer we-disease can emerge in response to acute, life-threatening and existential concerns experienced together as a couple, in vision impairment it may likely arise because of a long-term adaptation to constant readjustments in everyday life (e.g., balanced autonomy vs. dependence, role distribution) that affect both partners (Lehane et al., 2017). This assumption is supported by qualitative evidence suggesting that we-disease could also apply to the context of sensory loss, in which couples generally perceive their partner’s sensory impairment as a shared problem (“being in this together”; Mamali et al., 2020), and that couples adjust better, both individually and maritally, when they perceive the disability as “our problem” (Bertschi et al., 2021).

**Figure 1:**
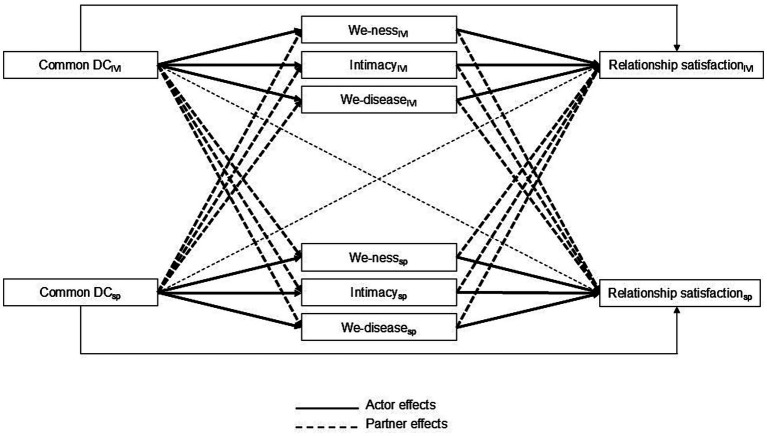
Conceptual diagram of the actor and partner indirect effects of we-ness, intimacy, and we-disease on the association between common dyadic coping and relationship satisfaction. Common DC as the independent variable, we-ness, intimacy, and we-disease as mediators, and relationship satisfaction as the dependent variable. Correlations between partners’ predictors, error variances of the mediators and outcome variables, as well as covariates (i.e., relationship length and marital status) were omitted from the figure for clarity. DC, dyadic coping; IVI, individual with vision impairment; SP, spouse.

The main aim of this study was to explore the mediating role of we-ness, intimacy, and we-disease on the relationship between common DC and relationship satisfaction, while accounting for the dyadic interdependence within couples facing vision impairment (see Figure 1). We hypothesized that higher engagement in common DC would lead to greater (a) perceptions of *we-ness* (as it would be in most couples regardless of vision impairment), (b) perceptions of *we-disease* (as vision impairment is a shared stressor and affects the couple as a whole), and (c) intimacy (as the impairment welds partners together). Improvements in these couple processes would, in turn, lead to higher levels of relationship satisfaction.

## Materials and methods

### Participants

The sample of the current study consisted of *N* = 99 different-gender couples (*N* = 198 individuals) (see Table 1). Individuals with vision impairment (IVI) were on average 59.41 years old (*SD* = 16.48) and spouses on average 59.56 years old (*SD* = 15.45). Of IVI, 52.5% identified as men and 47.5% as women. Approximately 40% of IVI and spouses had a university degree and around 40% had completed high school. While half of the spouses was working at the time of the study, approximately a third of IVI was paid for working. Couples were in the current relationship for an average of 31 years. Most couples were married and lived together without children in household. Regarding the vision impairment, around two thirds of IVI experienced a gradual decline (60.6%) and lived with vision impairment for 10 years or longer (67.7%). Most IVI (57.6%) felt highly impaired due to their vision impairment.

**Table 1:** Sample characteristics (*N* = 99 couples).

Variables	IVIs		Spouses	
	*M (range)*	*SD*	*M (range)*	*SD*
Age	59.41 (31–93)	16.48	59.56 (28–89)	15.45
Relationship length (years)	31.17 (2–69)	18.28	-	-
	*n*	%	*n*	%
Gender				
Men	52	52.5	47	47.5
Women	47	47.5	52	52.5
Education				
Middle school	13	13.1	15	15.2
High school	41	41.4	36	36.4
University	40	40.4	46	46.5
No information	5	5.1	2	2.0
Employment				
Full time	4	4.0	24	25.3
Part time	26	25.7	23	24.2
No information^a^	3	3.0	-	-
No paid work	23	22.8	9	8.9
Retirement	43	43.4	43	43.4
Marital status				
Married	83	83.8	-	-
Not married	16	16.2	-	-
No information	0	0	-	-
Co-habitation status				
Living together	90	90.9	-	-
Living apart	5	5.1	-	-
No information	4	4.2	-	-
Children in household				
Yes	27	27.3	-	-
No	72	72.7	-	-
No information	0	0	-	-
Vision loss onset				
Congenital stable	2	2.0	-	-
Gradual	60	60.6	-	-
Sudden / rapid deterioration	18	18.2	-	-
Mixed	13	13.1	-	-
No information	6	6.0		
Years since onset of vision loss				
≤ 4	15	15.2	-	-
5–9	17	17.2	-	-
≥ 10	67	67.7	-	-
Subjective level of impairment due to vision problems; *M* = 2.84; *SD* = 1.06, *Min-Max* = 0–4				
Not/slightly impaired	9	9.1	-	-
Moderately impaired	28	28.3	-	-
Highly impaired	62	57.6	-	-

IVI, individual with visual impairment; ^a^Employed but information missing regarding level of employment.

### Procedure

The present study used data from an initial baseline questionnaire of the Sensory Loss in the Dyadic Context (SELODY) study (Bodenmann et al., 2018). This project was approved by the Ethics Committee of the Faculty of Arts and Social Sciences at University of Zurich (approval 19.4.6). The rational of the SELODY study was to explore the impact of one partner’s sensory loss on the couple relationship, partners’ experience of stress, and how members of the couple cope with stress together.

Inclusion criteria for study participation were: (a) one partner having a visual impairment that had developed or significantly deteriorated during the current relationship, (b) to be over 18 years of age, and (c) to speak either French, German, or Italian. Between May and December 2019, we advertised the study in flyers, newsletters, magazines, and on social media channels addressing people with visual impairment. Additionally, eligible couples were introduced to the study by social workers at specified counseling centers in Switzerland. Interested couples were mailed information about the study and were asked to give their written consent prior to participation. Considering the specificities of the population, we offered various modes of participation (i.e., online survey, paper-pencil survey, or a telephone interview in which trained research assistants read the questions out to the participants who then gave their answers orally). Participants received no financial compensation for participation.

Of the 123 couples who showed interest in participation, eight did not return questionnaires at T1, *n* = 3 failed to meet the inclusion criteria for this study (both partners rather than one had a visual impairment), and *n* = 12 were excluded because only one partner completed the survey questionnaires (current sample: *N* = 99 couples included).

### Measures

Besides sociodemographic and vision impairment related information, the following self-report questionnaires were completed by each partner.

**Common dyadic coping.** We used the *common dyadic coping* subscale of the Dyadic Coping Inventory (DCI; Bodenmann, 2008). This subscale of the DCI assesses couple-oriented behaviors in which couples engage to cope jointly with stress. The scale comprises five items (e.g., “We engage in a serious discussion about the problem and think through what has to be done”), which are rated on a 5-point scale from 1 = *never/very rarely* to 5 = *very often*. Total score ranges between 1 and 5, with higher values representing higher engagement in common DC behaviors. The DCI showed good psychometric properties in the three official language groups of Switzerland (German, French, and Italian; Ledermann et al., 2010). Internal consistency in the current study was good (IVI: *ω* = 0.75; spouse: *ω* = 0.83).

**We-ness.** Participants completed the We-ness Questionnaire (WNQ; Vedes and Bodenmann, 2013). This scale contains 8 items that assess a couple’s sense of togetherness and the extent to which the partners have an “us/we” vs. a “I/me” orientation (e.g., “In our relationship, instead of two “I ‘s, there is one ‘We”). Participants rated these items on a 4-point scale from 0 = *not at all true* to 3 = *very true*. The total range varies from 0 to 3, and higher scores represent more we-ness. Reliability was good in the current sample (IVI: ω = 0.80; spouse: *ω* = 0.84).

**Intimacy.** We used four items to assess intimacy as defined by Debrot et al. (2012), which cover feelings of being secure, cared for, close to, and understood by the partner (e.g., “I feel close to my partner”). Participants rated these items on 5-point scales from 0 = *does not apply* to 4 = *applies very strongly.* Total score ranges between 0 and 4, and higher scores indicate more intimacy. Confirmatory factor analyses of the original version showed good model fit in both women and men, supporting the scale construct validity (Debrot et al., 2012). Internal consistency was very good in the current study (IVI: *ω* = 0.82; spouse: ω = 0.87).

**We-disease.** We measured we-disease with a newly developed We-Disease Questionnaire (WDQ; Vogt et al., 2024). This scale contains 4 items (e.g., “My visual impairment / My partner’s visual impairment is a shared challenge for us as a couple), which are rated on a 6-point scale from 0 = *not true at all* to 5 = *very true*. The WDQ demonstrated good internal consistency and correlated with measures of dyadic adjustment in the expected directions (Vogt et al., 2024). Internal consistency was satisfactory to good ranging between ω = 0.66 to 0.86 (Vogt et al., 2024). In the current study, reliability was acceptable (IVI: ω = 0.67; spouse: ω = 0.69).

**Relationship satisfaction.** For assessing relationship satisfaction, we selected the 4-item version of the Couples Satisfaction Index (CSI-4; Funk and Rogge, 2007). The CSI comprises four items (e.g., “In general, how satisfied are you with your relationship?”) to be answered in 6−/7-point response scale with various verbal anchors, like 0 = *not at all true* to 5 = *completely true*. A total score can be computed by summing up the scores on the 4 items (range 0–21). Higher scores indicate more relationship satisfaction. In the current study, reliability was very high (IVI: ω = 0.82; spouse: ω = 0.90).

Since the survey was available in three different language groups and official translations for each language were not always available (the DCI is the unique measure validated in the three official languages of Switzerland), we translated the scales into the target languages following a forward-backward translation approach to develop high-quality and accurate translations. Because the scales and corresponding items are brief and simple, the items were literally translated. The translated and original versions were compared to ensure semantic consistency. All translations and back-translations were carried out by native speakers. Finally, the translated versions were further pilot tested with a few numbers of participants from each language group to account for potential misunderstandings among respondents. No differences were found between the three language groups in any measures.

### Data analysis

Paired *t*-test and Pearson correlations were computed for descriptive statistics and associations between study variables, respectively, using IBM SPSS, version 28. Pearson correlations (for continuous variables) and independent *t*-tests (for categorical variables) between all sociodemographic variables and relationship satisfaction were computed to identify potential covariates (only variables significantly associated with relationship satisfaction were controlled for in subsequent analyses). An Actor-Partner Interdependence Mediation Model (APIMeM; Ledermann et al., 2011) was computed in Mplus, version 8 (Muthén and Muthén 1998–2017) to assess whether we-ness, intimacy, and we-disease mediated the relationships between common DC and relationship satisfaction. All variables were included in the same mediation model to allow for the evaluation of the unique contribution of each mediator. Bootstrap resampling procedures with 1,000 samples was computed to estimate statistical significance of the total, direct and indirect effects with a 95% bias-corrected confidence interval; when the confidence interval did not include zero, the effects were considered significant.

To perform the APIMeM, we followed the guidelines for dyadic data analysis, in which the predictor and mediators were centered around the grand-mean and unstandardized path coefficients and their standard errors were presented (Kenny et al., 2006). Additionally, to improve interpretation and comparison across coefficients, standardized coefficient regressions were also reported, after standardizing variables using mean and standard deviation computed across IVI and spouses (Kenny et al., 2006). To reach the most parsimonious models, we first analyzed whether actor and partner effects varied between IVI and spouses (i.e., are moderated by partner role). Accordingly, each pair of actor effects and partner effects, separately, were fixed as equal between IVI and spouses. The fit of this fully constrained model was analyzed with the qui-square test, considering *χ^2^* at *p* >. 05 as good model fit. In the presence of a non-significant qui-square, model misspecification was addressed by successively unconstraining paths coefficients and computing Δ*χ^2^*. A significant difference indicates that the paths are statistically different between IVI and spouses and should allow to vary freely. Overall, parameter estimates apply to both IVI and spouses. Parameter estimates that differ between partners were otherwise reported. Missing data were handled with the Full Information Maximum Likelihood method. The fit of the estimated models was interpreted accordingly: *χ^2^* with a significance >0.05; CFI ≥ 0.90, RMSEA ≤ 0.08, and standardized root-mean-square residual (SRMR) ≤ 0.10, as indicators of acceptable model fit; and CFI ≥ 0.95, RMSEA ≤ 0.05, and SRMR ≤ 0.08 as indicators of good model fit (Hu and Bentler, 1998). The magnitude of effect sizes was interpreted according to Cohen (1988) guidelines: small: *d* ≥ 0.20, *r* ≥ 0.10, *R*^2^ ≥ 0.02; medium: *d* ≥ 0.50, *r* ≥ 0.30, *R*^2^ ≥ 0.13; large: *d* ≥ 0.80, *r* ≥ 0.50, *R*^2^ ≥ 0.26. A significance level of *p* <. 05 was considered in all analyses.

## Results

### Descriptives and intercorrelations between study variables

Compared to their spouses, IVI reported higher levels of intimacy, we-ness, common DC, and relationship satisfaction. IVI and spouses reported similar levels of we-disease. IVI and spouses’ scores were moderately to strongly correlated, showing dyadic interdependence. Overall, study variables were significantly, positively, and moderately to strongly correlated with each other; smaller associations were found for we-disease (see Table 2).

**Table 2:** Descriptive statistics and bivariate correlations between study variables.

Variables	Descriptives				Correlations				
	IVI	Spouses	Diff_IVI-spouses_		1	2	3	4	5
	*M* (*SD*)	*M* (*SD*)	*t*	*d*					
Common DC	3.49 (0.74)	3.26 (0.82)	2.73**	0.29	0.43***	0.38***	0.56***	0.17	0.55***
We-ness	2.32 (0.46)	2.19 (0.49)	2.63*	0.27	0.43***	0.49***	0.61***	0.41***	0.56***
Intimacy	3.55 (0.55)	3.28 (0.68)	4.28***	0.44	0.53***	0.59***	0.54***	0.34***	0.78***
We-disease	3.15 (0.94)	3.14 (0.96)	0.15	0.01	0.17	0.37***	0.24*	0.29**	0.20*
Relationship satisfaction	16.97 (2.94)	15.85 (3.19)	3.19**	0.37	0.55***	0.53***	0.73***	0.17	0.36***

Correlations for IVI are presented below the diagonal, and for spouses above the diagonal. Correlations within couples are shown in bold on the diagonal. DC, dyadic coping; IVI, individual with vison loss. *p < 0.05; **p < 0.01; ***p < 0.001.

IVI’s relationship satisfaction was significantly associated with relationship length (*r* = −0.27, *p* = 0.007). Married IVI reported less relationship satisfaction than non-married IVI, *t*(97) = −2.28, *p* = 0.025. Accordingly, the effect of these variables on IVI’s relationship satisfaction was controlled for in the analyses. No significant associations were found between sociodemographics and spouses’ relationship satisfaction.

### Actor-partner interdependence mediation model

The fully constrained model yielded a qui-square of 33.59, *df* = 16, *p* = *0*.006. We unconstrained successively each path of coefficients and examined whether model fit would improve. Two partner effects and one actor effect were allowed to vary freely across IVI and spouses as the model fit improved significantly: the partner effect of we-ness on relationship satisfaction (Δ*χ^2^* = 11.26, Δ*df* = 1, *p* < *0*.001) and of we-disease on relationship satisfaction (Δ*χ^2^* = 17.23, Δ*df* = 1, *p* < *0*.001), and the actor effect of we-ness on relationship satisfaction (Δ*χ^2^* = 5.47, Δ*df* = 1, *p* = *0*.019). The model included two covariates, relationship length and marital status, given their significant associations with IVI’s relationship satisfaction. The final model fitted the data well: *χ^2^* = 5.97, *df* = 13, *p* = 0.947; RMSEA = 0.000; SRMR = 0.034; CFI = 1.000, and explains 66 and 71% of the variance of relationship satisfaction for IVI and spouses, respectively.

**Actor and partner effects between common DC and intimacy, we-ness and we-disease.** A significant positive actor effect between common DC and relationship satisfaction was found. Significant positive actor and partner effects between common DC and intimacy were observed, as well as a significant positive actor effect between common DC and we-ness. A significant positive partner effect between common DC and we-disease was observed (see Table 3).

**Table 3:** From common DC to relationship satisfaction: direct and total effects.

Effects	*B* (*SE*)	*β*
Actor direct effects		
Relationship length→Relationship satisfaction_ivi_	−0.04 (0.01)**	−0.23**
Marital status^a^→Relationship satisfaction_ivi_	−0.23 (0.54)	0.03
Common DC→Relationship satisfaction	0.78 (0.21)***	0.19***
Common DC→We-ness	0.23 (0.05)***	0.37***
Common DC→Intimacy	0.38 (0.06)***	0.49***
Common DC→We-disease	0.09 (0.16)	0.07
We-ness_ivi_→Relationship satisfaction_ivi_	0.42 (0.52)	0.06
We-ness_sp_→Relationship satisfaction_sp_	1.87 (0.49)***	0.30***
Intimacy→Relationship satisfaction	2.82 (0.44)***	0.53***
We-disease→Relationship satisfaction	−0.01 (0.12)	−0.00
Partner direct effects		
Common DC→Relationship satisfaction	0.01 (0.22)	0.00
Common DC→We-ness	0.06 (0.04)	0.10
Common DC→Intimacy	0.13 (0.05)*	0.19*
Common DC→We-disease	0.31 (0.14)*	0.26*
We-ness_ivi_→Relationship satisfaction_sp_	−0.88 (0.50)	−0.13
We-ness_sp_→Relationship satisfaction_ivi_	1.07 (0.55)^†^	0.18^†^
Intimacy→Relationship satisfaction	−0.05 (0.33)	−0.01
We-disease_ivi_→Relationship satisfaction_sp_	−0.76 (0.20)***	−0.23***
We-disease_sp_→Relationship satisfaction_ivi_	0.00 (0.16)	0.07
Actor total effect		
Common DC_ivi_→Relationship satisfaction_ivi_	1.99 (0.27)***	0.48***
Common DC_sp_→Relationship satisfaction_sp_	1.98 (0.31)***	0.51***
Partner total effect		
Common DC_ivi_→Relationship satisfaction_sp_	0.18 (0.30)	0.05
Common DC_sp_→Relationship satisfaction_ivi_	0.62 (0.24)**	0.17**

Unstandardized and standardized maximum likelihood estimates are displayed. For parameter estimates set as equal for IVI and spouses, only one estimate is presented. Parameters that differ between IVI and spouses are reported accordingly. Significant estimates are in bold. DC, dyadic coping; IVI, individual with vison impairment; SP, spouse. R^2^, Relationship satisfaction: IVI = 0.66, spouses = 0.71; R^2^ We-ness: IVI = 0.18, spouses = 0.18; R^2^ Intimacy: IVI = 0.36, spouses = 0.29; R^2^ We-disease: IVI = 0.09, spouses = 0.00. †*p* < 0.10; *p < 0.05; **p < 0.01; ***p < 0.001. ^a^Reference group: 0 = Not married.

**Actor and partner effects between we-ness, intimacy, and we-disease and relationship satisfaction.** A significant positive actor effect between intimacy and relationship satisfaction was observed among both IVI and spouses, as well as between we-ness and relationship satisfaction but only among spouses. Two significant partner effects showed that (1) spouses’ higher perceptions of we-ness were marginally related to IVI’s higher levels of relationship satisfaction (*p* = 0.05) and (2) IVI’s higher perceptions of we-disease were significantly associated with spouses’ lower levels of relationship satisfaction (see Table 3).

**Figure 2:**
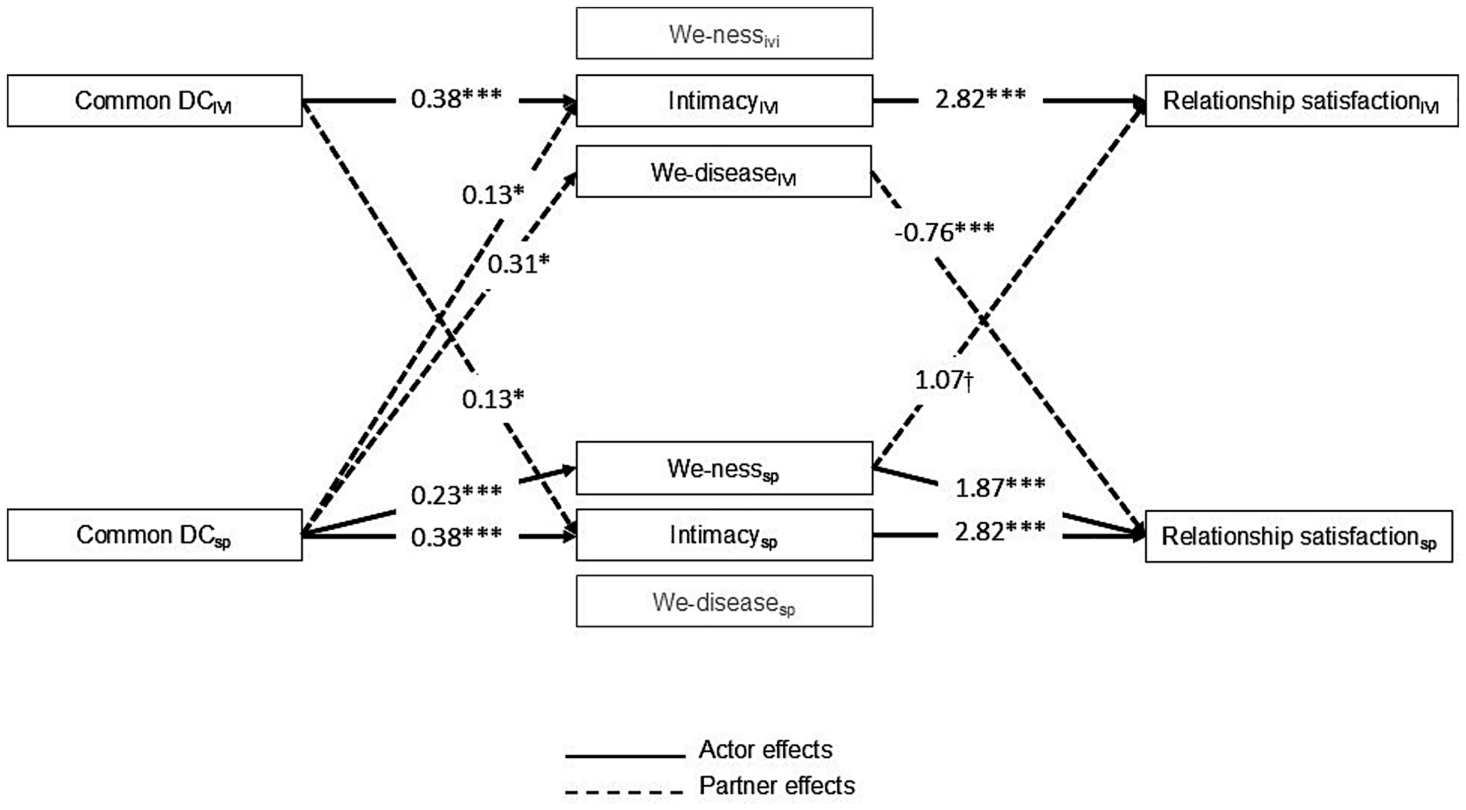
Diagram of the statistically significant indirect effects of we-ness, intimacy, and we-disease on the associations between common dyadic coping and relationship satisfaction. For clarity, only significant indirect effects were presented. Paths values represent unstandardized regression coefficients. Relationship length and marital status were controlled for in the model. DC, dyadic coping; IVI, individual with vision impairment; SP, spouses. †*p* < 0.10; *p < 0.05; **p < 0.01; ***p < 0.001.

**Indirect effects of we-ness, intimacy, and we-disease on the associations between common DC and relationship satisfaction.** Beyond the direct effects described above, we found that common DC was indirectly related to relationship satisfaction through intimacy, we-ness, and we-disease. Specifically, as displayed in Figure 2, the results showed that (1) higher levels of common DC were associated with higher levels of intimacy which, in turn, were associated with higher levels of relationship satisfaction; (2) the more one partner engages in common DC, the more the other partner’s perceived intimacy and, consequently, the more they are satisfied with the relationship; (3) the more spouses engage in common DC, the more they perceived we-ness and, consequently, the more they and IVI are satisfied with the relationship; and (4) the more spouses engage in common DC, the more IVI perceived we-disease and, consequently, the less spouses are satisfied with the relationship (*actor-partner-actor*; see Table 4).

**Table 4:** Indirect effects of we-ness, intimacy, and we-disease on the associations between common dyadic coping (DC) and relationship satisfaction (RS).

Indirect effect	IE (*SE*)	*p*	95%CI (LLCI/ULCI)
Effects from IVI’s Common DC to IVI’s RS			
Common DC_ivi_→We-ness_ivi_→RS_ivi_	0.10 (0.13)	0.452	[−0.15, 0.37]
Common DC_ivi_→We-ness_sp_→RS_ivi_	0.06 (0.06)	0.324	[−0.01, 0.24]
Common DC_ivi_→Intimacy_ivi_→RS_ivi_	**1.06 (0.24)**	**<0.001**	**[0.67, 1.59]**
Common DC_ivi_→Intimacy_sp_→RS_ivi_	−0.00 (0.04)	0.886	[−0.10, 0.07]
Common DC_ivi_→We-disease_ivi_→RS_ivi_	−0.00 (0.03)	0.980	[−0.14, 0.03]
Common DC_ivi_→We-disease_sp_→RS_ivi_	0.00 (0.03)	0.981	[−0.02, 0.13]
Effects from Spouses’ Common DC to IVI’s RS			
Common DC_sp_→We-ness_ivi_→RS_ivi_	0.02 (0.04)	0.527	[−0.02, 0.14]
Common DC_sp_→We-ness_sp_→RS_ivi_	**0.25 (0.14)**	**0.073**	**[0.02, 0.54]**
Common DC_sp_→Intimacy_ivi_→RS_ivi_	**0.36 (0.16)**	**0.027**	**[0.08, 0.73]**
Common DC_sp_→Intimacy_sp_→RS_ivi_	−0.02 (0.13)	0.885	[−0.26, 0.26]
Common DC_sp_→We-disease_ivi_→RS_ivi_	−0.00 (0.02)	0.918	[−0.08, 0.01]
Common DC_sp_→We-disease_sp_→RS_ivi_	0.00 (0.03)	0.994	[−0.01, 0.19]
Effects from Spouses’ Common DC to spouse’s RS			
Common DC_sp_→We-ness_sp_→RS_sp_	**0.43 (0.16)**	**0.008**	**[0.17, 0.78]**
Common DC_sp_→We-ness_ivi_→RS_sp_	−0.05 (0.05)	0.303	[−0.18, 0.01]
Common DC_sp_→Intimacy_sp_→RS_sp_	**1.06 (0.24)**	**<0.001**	**[0.67, 1.69]**
Common DC_sp_→Intimacy_ivi_→RS_sp_	−0.01 (0.04)	0.886	[−0.10, 0.07]
Common DC_sp_→We-disease_sp_→RS_sp_	−0.00 (0.03)	0.980	[−0.14, 0.01]
Common DC_sp_→We-disease_ivi_→RS_sp_	**−0.23 (0.12)**	**0.052**	**[−0.61, −0.06]**
Effects from IVI’s Common DC to Spouse’s RS			
Common DC_ivi_→We-ness_sp_→RS_sp_	0.11 (0.09)	0.218	[−0.04, 0.32]
Common DC_ivi_→We-ness_ivi_→RS_sp_	−0.20 (0.13)	0.119	[−0.50, 0.02]
Common DC_ivi_→Intimacy_sp_→RS_sp_	**0.36 (0.16)**	**0.027**	**[0.08, 0.73]**
Common DC_ivi_→Intimacy_ivi_→RS_sp_	−0.02 (0.13)	0.885	[−0.26, 0.26]
Common DC_ivi_→We-disease_sp_→RS_sp_	−0.00 (0.02)	0.918	[−0.08, 0.01]
Common DC_ivi_→We-disease_ivi_→RS_sp_	−0.07 (0.13)	0.583	[−0.34, 0.20]

Unstandardized maximum likelihood estimates for indirect effects (IE) are displayed. Significant IE are in bold. Relationship length and marital status were controlled for in the model. CI, confidence interval; DC, dyadic coping; LLCI/ULCI, lower and upper CI; RS, relationship satisfaction; IVI, individual with vison impairment; SP, spouse.

**Separate mediation models**. Because mediators were moderately to strongly intercorrelated, separate models with each mediator at a time were conducted to account for concerns associated with multicollinearity. The models for intimacy and we-disease did not yield substantial differences from the model with all mediators included together. Regarding the model for we-ness, contrary to the full model, the effect of IVI’s we-ness on their own (*B* = 1.77, *p* = 0.002) and their spouses (*B* = −1.90, *p* = 0.007) relationship satisfaction was statistically significant. Two additional indirect effects were found: the more IVI engage in common DC, the more they perceived we-ness (*B* = 0.23, *p* < 0.001) and, consequently, the more they are satisfied with the relationship (*B* = 1.77, *p* = 0.002; IE = 0.40, 95% CI [0.13, 0.78]), but the less their spouses are satisfied with the relationship (*B* = −1.90, *p* = 0.007; IE = −0.43, 95% CI [−0.82, −0.13]).

## Discussion

This study highlighted unique pathways from common DC to relationship satisfaction among couples in which one partner faces vision impairment based on each we-process.

The direct effects of common DC on intimacy and we-ness support our hypotheses that joint DC strategies are likely to enhance partners’ sense of togetherness/unit (“we-ness”), as well as proximity and attachment between them (intimacy), one of the main functions of DC (Bodenmann, 2005; Bodenmann et al., 2016; Cutrona, 1996). This consequently is associated with partners feeling more satisfied with their relationship. Yet, distinct pathways from we-ness and intimacy to relationship satisfaction emerged between partners.

Specifically, regarding intimacy, our findings expanded empirical backing to Laurenceau et al.’s (1998) interpersonal model of intimacy, by showing how satisfied relationships, as perceived by both IVI and spouses, benefitted from *mutual* responsiveness (i.e., complementary engagement in DC responses), as this seems to help couples to feel understood, close, cared for, validated, and safe in their committed relationship. Our findings are also in line with previous research that showed how shared activities (e.g., that lead to self-expansion) promote relationship quality and intimacy (Laurenceau et al., 2004). Also, of note is that the benefits of common DC for intimacy were not only observed *within-person* but also *across partners*, which may indicate that the benefits of this form of DC extend beyond individual’s perceptions.

Regarding we-ness, our results mirror prior research that a shared identity in a relationship (Reid et al., 2006) is likely to be enhanced when partners jointly engage in coping behaviors when dealing with dyadic stress (Aydogan et al., 2024; Topcu-Uzer et al., 2021), and that we-ness contributes to relationship satisfaction (e.g., Cruwys et al., 2022; Nobandegani, 2022; Özgülük Üçok et al., 2024; Reid et al., 2006). This is in line with the qualitative findings of Bertrand et al. (2022) illustrating partners’ efforts to maintain their sense of we-ness through reshaping their conjoint engagement in everyday activities when coping with one partner’s vision impairment. It is interesting to note that even though IVI hold greater perceptions of we-ness, the pathways between common DC to relationship satisfaction via we-ness only occurred through spouses’ perceptions (when all three mediators are included together in the same model). This suggests that the benefits of perceiving the relationship as a cohesive unit are more consistent when such perceptions are held by spouses. Since vision impairment may strain the couple relationship, namely by contributing to role inequities, being a risk for separation (Lehane et al., 2017), when spouses hold a sense of we-ness/togetherness in their relationship, this could be interpreted as a sign of spouses’ investment in, and commitment with, the relationship (Afifi et al., 2016), which further benefits both partners, especially for IVI. However, we should note that in the model where only we-ness was included as a potential mediator, IVI’s own engagement in common DC also contributed to their relationship satisfaction via one’s own sense of we-ness, suggesting that the role of their own perceptions of togetherness could be mitigated by other, more relevant we-processes for their relational adjustment. Likewise, a negative effect of IVI’s sense of we-ness on their spouses’ relationship satisfaction emerged and could be interpreted along with the effect of IVI’s perceptions of we-disease on spouses, discussed below.

Regarding we-disease, couples perceived similar levels of we-disease, supporting the conceptualization of vision impairment as a shared, interpersonal experience within couples (Bertschi et al., 2021), similarly to what was found in the context of one partner’s chronic disease (e.g., Kayser et al., 2007; Leuchtmann and Bodenmann, 2017). Accordingly, the multifaceted consequences of vision loss seem to affect both partners, which are likely to reinforce a we-perspective. This is aligned with theoretical assumptions of the STM (Bodenmann, 2005; Bodenmann et al., 2016). However, while theoretical models suggest that dyadic appraisals are an implicit component of common DC and are related to dyadic adjustment (Bodenmann et al., 2016; Helgeson et al., 2018; Kayser et al., 2007), in our study, perceiving vision impairment as we-disease was hardly associated with either common DC or relationship satisfaction. Still, IVI’s perceptions of we-disease seem to account for the relationship between spouses’ common DC and their relationship satisfaction.

First, regarding the first path of this mediation model, our results suggested that when spouses engaged in common DC, IVI but not spouses are likely to consider vision impairment as a shared problem. This suggests that joint efforts to cope with stress have interpersonal benefits that help strengthen a sense of vision impairment as a shared stress in the other partner (in this case, IVI, who is perhaps more in need to feel that they are not alone in the coping process with their disabling condition). As a possible explanation for our findings, it may be that for the IVI, perceiving their disabling condition as a we-disease plays a more important role, as they could perceive that their impairment may burden the partner. Therefore, IVI may be more aware of the impact on the couple as a whole. This awareness may be fueled by their experience that they are the receiver of common DC (as demonstrated by the positive partner effect from spouses’ common DC to IVI’s we-disease).

Regarding the second path of this mediation model, our findings suggest that higher shared appraisals of vision impairment is not always beneficial for couple’s relationship satisfaction, with either no impact (for IVI) or a negative one (for spouses). This is contrary to prior qualitative studies suggesting that being “in it together” was beneficial for couples’ adjustment to sensory-impairment related challenges (Mamali et al., 2020). One possible explanation for our findings is that cognitively appraising vision impairment as a shared stressor for both partners means that they also share coping responsibility (e.g., Helgeson et al., 2018; Kayser et al., 2007). Particularly for spouses, even so that engaging in joint coping strategies appears to be beneficial for their relationship satisfaction, dyadic appraisals could enhance their sense of responsibility for IVI’s well-being, as well as role overload (i.e., appraising the strain from caregiving as too high), which are primary stressors for spouses taking the role of caregiver (Pearlin et al., 1990). In our study, this seems to be particularly pronounced when IVI recognized vision impairment as a dyadic stressor. When IVI hold strong dyadic appraisals of the illness, they also expect a shared management of the illness. This may lead to IVI being more willing and comfortable to engage in common DC; however, consequently, spouses may feel burdened and distressed by their involvement in the illness (Helgeson et al., 2018). Similar findings were found in some studies with couples facing one partner’s diabetes, showing that shared appraisals of the illness could impair the healthy partner psychological functioning (e.g., Helgeson et al., 2022). Accordingly, it is plausible to assume that IVI’s expectations that spouses “are in it together” cause additional burden and distress for spouses. This rational can be supported by spouses reporting lower levels of couple satisfaction than IVI, underlying the negative impact of the partner’s vision impairment on the spouse’s satisfaction with the relationship (e.g., Strawbridge et al., 2007).

It may also be plausible to assume that the role of dyadic appraisals may presumably be more pronounced and adaptive in life-threatening health conditions, such as cancer, compared to disabling but not imminent life-threatening health impairments, such as vision impairment. Perhaps in the context of vision impairment, efforts to maintain or rebuild some level of autonomy is particularly adaptive for spouses. Indeed, even though the increased closeness and proximity promoted by conjoint coping efforts was related to better relationship satisfaction for both partners, dyadic appraisals may particularly force dependencies on each other, which can collide with a need of independence; this reflects an ambivalence that is common in couples facing one partner’s disabling health impairment (Bertschi et al., 2021). Specifically, in our study, IVI’s dyadic appraisals could exacerbate spouses’ perceptions of impairment of self and autonomy, who could already be struggling with their impairment of independence. In fact, dependency-related issues have also been reported as a major difficulty by spouses of individuals with sensory loss (Bertrand et al., 2022; Lehane et al., 2017; Mamali et al., 2020). This possible explanation can be supported by the similar potential downside of IVI’s greater perceptions of general unity and togetherness (we-ness) on spouses’ relationship satisfaction that emerge when we-ness is considered as a single mediator. While spouses’ sense of we-ness seems to be beneficial, when the partner with the disability perceives the couple as a unit in a broader sense, this could be detrimental for spouses, perhaps because it challenges dependence-autonomy issues, which become even more salient when we-disease perceptions are considered in the model.

## Strengths and limitations

A major strength of the current study is the focus on vision loss as a dyadic experience, thus being able to simultaneously test interdependencies. Several limitations need to be considered when interpreting the results. First, test power may be limited by the relatively small sample size. However, we still tested multiple variables simultaneously as a starting point to stimulate more research in this context. On a related note, while this study is specific to different-sex couples facing one partner’s vision impairment, the results may inform broader contexts and inspire future studies. For instance, they seem to contribute to affirm shared illness appraisals as less advantageous for healthy partners, a pattern that was previously suggested in the context of diabetes (e.g., Helgeson et al., 2022). Future studies would shed some light on whether these findings are more context-specific or more general across different contexts, including same-sex couples. Secondly, relying on cross-sectional and self-report data inhibits causal interpretations. Indeed, the associations between common DC, we-processes, and relationship satisfaction are likely to be bidirectional, and couple processes may be more accurately captured through daily-diary methods (e.g., Hou et al., 2025). Thus, future studies using longitudinal cross-lagged panel models and including other data sources, like diaries or observational data from standardized laboratory interactions, are warranted. On a related note, additional contextual information about vision impairment, such as its cause (e.g., genetic vs. accident), should be collected in further research, since it could influence how couples appraise, emotionally react to, and cope with vision impairment. Third, the sample was quite homogenous, heterosexual, predominantly well-educated, and living with the visual impairment for several years already. Additionally, beyond the low reliability of the we-disease scale discussed above, overall, study measures were moderately reliable. Another instrument-related issue is that most of the measures used in the present study have not been previously validated through rigorous psychometric studies, even less in the three language groups. Furthermore, the findings of this study should be interpreted considering those limitations.

## Conclusions and implications for health psychology

Common DC was related to relationship satisfaction more strongly via we-ness and intimacy than we-disease in the context of vision impairment. This suggests that this form of DC helps to promote positive aspects of the relationship quality that are not necessarily related to the health impairment (i.e., acknowledging it as “our problem”), but are meaningful for couples facing one partner’s vision impairment. From a clinical standpoint, healthcare and rehabilitation settings should help couples affected by one partner’s vision impairment to be aware of the importance of and to engage in conjoint strategies to cope with stress, since they seem to promote couples’ sense of togetherness and intimacy, with benefits for couples’ relational adjustment to the stressors associated with vision impairment. This could be achieved through well-established programs (e.g., Couples Coping Enhancement Training [CCET]; Bodenmann and Shantinath, 2004).

Particularly, our findings emphasize that fostering we-ness and intimacy may be more protective than shared appraisals of vision impairment, especially for spouses at risk for greater burden. In the context of psychosocial rehabilitation, prevention strategies may then focus on enhancing open communication and self-disclosure and training both partners in active listening and responsiveness (to achieve greater intimacy), along targeting shared goals and narrative (to achieve greater we-ness). Existing evidence-based interventions targeting such relationship processes among couples dealing with one partner’s health condition (e.g., the Intimacy Enhancing Couples’ Therapy [IECT]; Manne and Badr, 2008) could be further adapted to the specific context of vision impairment.

Our findings highlighted that the adaptiveness of dyadic appraisals may not be universal across different health contexts and may have a different meaning (a) in the context of different mental disorders and physical illnesses (e.g., cancer, arthritis, heart diseases, rheumatism) and disabilities (e.g., vision or hearing impairment, physical handicap), (b) depending on the stage of the illness, (c) the duration of exposure to the stressor and (d) presumably other variables (e.g., available resources, commitment, own well-being, previous experiences of provision and reception of DC). Accordingly, its relevance for the psychosocial adjustment of couples facing certain forms of disabling health conditions should be further explored. Particularly, our findings suggest that dyadic appraisals of vision impairment can presumably affect the balance of autonomy versus interdependence of spouses of IVI. Accordingly, the extent to which couples negotiate cognitive representations of vision impairment, as well as boundaries at an individual and relational level, should be considered in psychosocial rehabilitation interventions. At a certain stage, it would be advisable to guide IVI’s spouses in disengaging from the shared illness identity to preserve their psychological and relational well-being. This process should also include IVI, not only to foster their awareness of the potential downsides that a conjoint view of the illness can have on their spouses, but also to promote shared understanding and collaboration. Such joint approach should be considered a critical component of psychosocial interventions targeting couples coping with vision impairment.

In sum, our findings shed light on the complexity to the process of stress and coping among couples facing vision impairment: while they suggest that intervention efforts should target symmetrical engagement in joint coping strategies, as well as the enhancement of we-ness and intimacy, they should consider not neglect each partner’s balance of autonomy and interdependence when coping with disability.

## Data Availability

The raw data supporting the conclusions of this article will be made available by the authors, without undue reservation.
